# The immersive experience of virtual reality during chemotherapy in patients with early breast and ovarian cancers: The patient’s dream study

**DOI:** 10.3389/fonc.2022.960387

**Published:** 2022-09-30

**Authors:** Alessandra Fabi, Luana Fotia, Federico Giuseppini, Arianna Gaeta, Chiara Falcicchio, Gabriele Giuliani, Antonella Savarese, Emanuela Taraborelli, Valentina Rossi, Paola Malaguti, Diana Giannarelli, Patrizia Pugliese, Francesco Cognetti

**Affiliations:** ^1^ Medical Oncology 1, Regina Elena National Cancer Institute, IRCCS, Rome, Italy; ^2^ Service of Psyco-Oncology, Regina Elena National Cancer Institute, IRCCS, Rome, Italy; ^3^ Twiceout, Rome, Italy; ^4^ Breast Unit, San Camillo Forlanini Hospital of Rome, Rome, Italy; ^5^ Biostatistic Unit, Regina Elena National Cancer Institute, IRCCS, Rome, Italy; ^6^ Department of Clinical and Molecular Medicine, University La Sapienza, Rome, Italy

**Keywords:** virtual reality, breast cancer, ovarian cancer, anxiety, chemotherapy, perceived time

## Abstract

**Background:**

A virtual reality experience (VRE) could represent a viable non-pharmacological intervention to reduce and better manage the main factors of psychophysical distress related to the diagnosis and treatment of cancer.

**Aim:**

The *“Patient’s Dream”* study was a two-arm randomized controlled trial conducted at the Regina Elena National Cancer Institute – IRCCS (Rome, Italy) from April 2019 to January 2020 to evaluate VRE impact in patients affected by breast or ovarian cancer. Before starting the first cycle of chemotherapy (CT), patients were randomized to receive the VRE (VRE arm) as “distraction therapy” or to entertain themselves with conventional means (control arm). The primary aims were the assessment of psychological distress, anxiety and quality of life between the two study arms. Secondary endpoints were the perceived time during the first course of CT and the acute and late toxicity.

**Results:**

Fourty-four patients were enrolled, 22 patients were randomly assigned to the VRE arm and 22 to the control arm. Collected data underline the absence of prevalent disturbs of anxiety and depression in both groups. Nevertheless, even if the state anxiety values before and after CT decreased in both groups, this reduction was statistically significant over time only in the VRE arm. The duration of therapy perceived by patients undergoing distraction therapy was significantly shorter when compared to the control group. The use of VRE during the first CT cycle appeared to reduce asthenia outcomes.

**Conclusion:**

Obtained data suggest that the VRE positively influenced the levels of state anxiety among cancer patients and support the continuous research on VRE as a distraction intervention, with the aim to meet the clinical need for effective nonpharmacologic adjunctive therapies.

**Clinical trial registration:**

https://clinicaltrials.gov/ct2/show/NCT05234996, identifier NCT05234996.

## 1 Introduction

The diagnosis of neoplastic disease is accompanied by an emotional complex process characterized by anxiety, depression, anger, and uncertainty about the present and future (1). The proposed treatments often cause anxiety and psychological distress further because of the toxicity profile and the frequent requirement for painful procedures (venipuncture, central venous access, invasive investigations) (2). Therefore, efforts to provide interventions to alleviate symptoms related to chemotherapy are an important area of research and improvement.

Evidence from the literature shows that an immersive virtual reality experience (VRE) can reduce procedural pain and anxiety in patients undergoing medical procedures, such as wound care or physical therapy for burn wounds, dressing changes for trauma injuries, procedures under local anesthesia, such as episiotomy repair and orthopedic surgery (3, 4). Indeed, virtual reality creates a sense of absorption in the virtual environment through special glasses and motion sensors, thus estranging the user from reality.

Based on these considerations and given the need for an integrated approach to managing cancer patients, VRE could represent a viable non-pharmacological intervention to reduce and better drive the main factors of psychological distress related to the diagnosis and treatment of cancer.

In the field of oncology, VRE can represent a “distraction therapy” tool that helps the cancer patient overcome physical limits and/or mental dictated by the disease condition (4, 5). This approach could also promote greater adherence to treatment with potential benefits on effectiveness and increase the host healthcare facility’s confidence and approval rating.

The “Patient’s Dream” was a prospective study designed to evaluate VRE impact in patients affected by breast or ovarian cancer. The present work assessed the improvement of psychological distress, anxiety and quality of life after a “distraction therapy” intervention by means of VRE, utilized during the first cycle of adjuvant chemotherapy (CT). The time perception by the patients during the treatment and acute and late toxicity were also assessed.

## 2 Patients and methods

### 2.1 Study design and setting

The “Patient’s dream” study is a two-arm randomized controlled trial conducted at Regina Elena National Cancer Institute – IRCCS (Rome, Italy) from April 2019 to January 2020; no stratification factors were planned. Before starting the first cycle of chemotherapy, patients were randomized to receive the VRE (VRE arm) as “distraction therapy” or to entertain themselves with conventional means (control arm), such as listening to music, watching a TV program, reading newspapers, books, magazines or also doing nothing, according to the patient’s preferences and for the entire duration of administration of the first CT cycle. A clinical team composed of three oncologists, three psychologists, one nurse and one expert VR operator supported the patients involved in the study.

The primary aims were the assessment of psychological distress, anxiety and quality of life between the two study arms. Secondary endpoints were the perceived time during the first course of CT and the acute and late toxicity.

The study was conducted in accordance with the ethical standards as laid down in the Declaration of Helsinki and its later amendments and within the protocol approved by the Central Ethics Committee (protocol registration number: RS 1105/18). Written informed consent was obtained from all participants included in the study. Clinical trial registration number: NCT05234996.

### 2.2 Patients

To be eligible for the study, all patients had to have a confirmed histological diagnosis of breast or ovarian cancer stage I–III, surgery as the first therapeutic approach, and be suitable to receive the first cycle of adjuvant CT, with or without a biological treatment according to specific cancer (regimens including anthracyclines/taxanes, anthracyclines/cyclophosphamide, carboplatinum/taxane, taxane alone combined or not with trastuzumab for breast cancer, carboplatin/paclitaxel combined or not with bevacizumab for ovarian cancer). Patients must be aged ≥18 years, with a median Eastern Cooperative Oncology Group (ECOG) performance status of 0–2, life expectancy >12 months and ability to understand and sign the informed consent. Patients presenting a previous history of alcohol and/or drug addiction, disorder of vision and eyes, and a history of psychiatric pathologies were not eligible.

### 2.3 Study evaluations

In both study arms, patients were evaluated as follows: before the start of the first infusion of CT (T1) with the Hospital Anxiety and Depression scales (HADs), the State–Trait Anxiety Inventory for Adults (STAI) in Y1 forms for the State Anxiety and Y2 for the Trait Anxiety and with the European Organization for Research and Treatment of Cancer quality of life questionnaire, Core 30 (EORTC QLQ-C30); immediately at the end of the infusion (T2) with STAI Y1 and with the investigation of the perceived time; within 48 h from the first CT cycle (T3) with HADs, STAI Y1, EORTC QLQ-C30; 1 week after the first CT cycle (T4) with a patient-reported outcomes (PROs) questionnaire; within 48 h from the second cycle (T5) with STAI Y1 and PROs questionnaire (**Table 1**). The description of each evaluation tool is provided in the following sections.

**Table 1:** Timeline of study measures.

	T1	T2	T3	T4	T5
HADs	X		X		
STAI Y1	X	X	X		X
STAI Y2	X				
Perceived Time		X			
EORTC QLQ-C30	X		X		
PROs				X	X

T1: before the start of the first infusion of CT; T2: immediately at the end of the infusion; T3: within 48 h from the first CT cycle; T4: one week after the first CT cycle; T5: within 48 h from the second CT cycle. HADs, Hospital Anxiety and Depression scales; STAI, State-Trait Anxiety Inventory for Adults; Y1 forms for the State Anxiety, Y2 for the Trait Anxiety; EORTC QLQ-C30, European Organization for Research and Treatment of Cancer quality of life questionnaire, Core 30; PROs, patient-reported outcomes.

#### 2.3.1 Psychological evaluations

##### 2.3.1.1 Psychological distress

The psychological distress was evaluated with the Hospital Anxiety and Depression scale (HADs) (6). The HADs is composed of 14 items, seven assess the anxiety status, and seven assess the depression status. The answers were given on a 4-point Likert scale (from 0 to 3) with a maximum of 21 points for anxiety and depression. A score ≥8 is the cut-off indicating the presence of anxiety or depression disorder. According to Carrol and collaborators, scores between 0 and 7 indicate a normal condition, scores between 8 and 10 indicate borderline cases, while scores ≥11 identify clinical cases (7).

##### 2.3.1.2 Anxiety

Anxiety has been evaluated with the STAI (6) in Y1 forms for the State Anxiety and Y2 for the Trait Anxiety. Each form consists of 20 items answered on a 4-point Likert scale (from 0 to 3). The final score ranges from 20 to 60, higher score corresponds to major anxiety.

#### 2.3.2 Quality of life assessment

Health-related quality of life (HRQoL) was assessed with EORTC QLQ-C30 Version 3.0 [eortc.org]. The questionnaire is made up of 30 items divided into 15 scales: Physical Functioning (PF), Role Functioning (RF), Social Functioning (SF), Emotional Functioning (EF), Cognitive Functioning (CF), Global QOL (QL), Fatigue (FA), Pain (PA), Nausea/Vomiting (NV), Appetite Loss (AP), Dyspnea (DY), Sleep Disturbances (SL), Diarrhea (DI), Constipation (CO), and Financial Impact of Disease (FI) (8). The score of each scale was obtained by a sum and a linear transformation and ranged from 0 to 100. In the functional scales, a higher score corresponded to better functioning of the area; in the symptomatic scales, a higher score corresponded to the worst of symptoms.

#### 2.3.3 Effective time and perceived time

The effective time was defined as the time from the start to the end of chemotherapy infusion; the nurse of the study team checked this time. The perceived time was the time felt and reported by the patient from the start to the end of CT infusion.

#### 2.3.4 Toxicity assessment

Toxicity was evaluated according to the Common Terminology Criteria for Adverse Events (CTCAE) v4.0 (9).

#### 2.3.5 Patient-reported outcomes questionnaire

Before the CT infusion, the oncologist illustrated and delivered the PRO-CTCAE™ questionnaire to the patient to be reported on subsequent visits (10). It was composed of 124 items and covered 78 symptoms. Symptoms evaluated can be detected by one up to a maximum of three characteristics: presence (yes; no); frequency (never; rarely; occasionally; frequently; almost constantly); severity (none; mild; moderate; severe; very severe); and/or interference with usual or daily activities (not at all; some; a bit; a lot; very). Some PRO-CTCAE™ symptoms comprise only one, while others include two, and some include three characteristics.

### 2.4 Distraction therapy modalities

The VRE was administered using three VR headsets containing a selection of audiovisual productions made with 360° technology and selected based on content, plot and production dynamics. During the entire experience, an operator dedicated to patient care was present to allow the most comfortable experience possible.

Patients were trained on the functioning of the headset, their interface, and how to select the preferred contents. Once familiar with the controls, the operator equipped the patients with high-quality audio headphones to complete the immersive effect of the contents. At that point, the patients were free to use the contents in complete autonomy. In any case, the operator remained close to the patient for the entire duration of the experience, giving advice on the contents or on the commands to be used from time to time. The contents, some created for the occasion and others made available by audiovisual production companies specialized in 360° videos, were carefully selected based on pre-established criteria:

- High viewing comfort (no abrupt camera movement, low risk of nausea, clear images etc.);

- Relaxing and engaging content, such as concerts, walks in the European capitals, mountain nature trails, isolated and fascinating places, pristine, exotic beaches, and Yoga sessions;

- Duration and comfortable viewing time, never more than 10 minutes for single content (the patient chose 3 or 4 contents).

The administration of VRE began with the therapy and lasted a maximum of 60–90 minutes. The VR headset used to provide such experiences was Oculus Go, a standalone VR headset developed by Facebook Technologies in partnership with Qualcomm and Xiaomi. The Oculus Go was an all-in-one headset and did not need to be tethered to an external device to use. It was equipped with a Qualcomm Snapdragon 821 chipset and a single 5.5-inch LCD with a resolution of 1280 × 1440 pixels per eye and a refresh rate of 72 or 60 Hz, depending on the application. The headset used Fresnel lenses that were improved over those used in the company’s previous headset, the Oculus Rift. It provided a field of view of about 101°, which gives the Go a display fidelity of 12.67 pixels per degree. Inputs were provided with a wireless controller that functions much like a laser pointer. The headset and controller utilized non-positional 3-degrees-of-freedom tracking, making it capable of seated or static-standing activities but unsuitable for room-scale applications.

### 2.5 Statistical analysis

The primary endpoint was the HADs scale. A sample size of 44 patients was needed to test an effect size (standardized mean difference among the 2 groups) of at least 0.70 considering a correlation of 0.50 and using repeated measurement analysis of variance (ANOVA) as a model. This sample size was determined to ensure a power of 80% at a significance level of 5%. Data were reported as mean and standard deviations, and the Student’s t-test was used to compare mean values. The chi-square test assessed associations between categorical variables. All analyses were performed using the IBM-SPSS statistical software, version 22.0. No adjustments for multiple tests were made.

## 3 Results

A total of 44 patients were enrolled; 22 patients were randomly assigned to the VRE arm and 22 to the control arm. The characteristics of the patients are shown in **Table 2**.

**Table 2:** Patient characteristics.

	VR arm (n = 22), n (%)		Control arm (n = 22), n (%)	
	Breast cancer, 17 (77.2%)	Gynecological cancer, 5 (22.7%)	Breast cancer, 19 (86.4%)	Gynecological cancer, 3 (13.6%)
Age (years), median (range)	51 (37–71)	50 (36–61)	50 (39–69)	52 (51–62)
Menopausal status:				
Pre Post	8 (47.1) 9 (52.9)	0 (0) 5 (100)	12 (63.2) 7 (36.8)	0 (0) 3 (100)
*BRCA* status				
Mutation Wild-type Not done	1 (5.9) 10 (58.8) 6 (35.3)	1 (20) 3 (60) 1 (20)	0 (0) 5 (26.3) 14 (73.7)	1 (33.3) 1 (33.3) 1 (33.3)
ECOG performance status:				
0 1	16 (94.1) 1 (5.9)	4 (80) 1 (20)	18 (94.7) 1 (5.2)	2 (66.6) 1 (33.3)
Hormonal receptors:				
Negative Positive	4 (23.5) 13 (76.5)	0 (0) 0 (0)	6 (31.6) 13 (68.4)	0 (0) 0 (0)
HER2:				
Negative 2+/FISH+ 3+	15 (88.2) 0 (0) 2 (11.8)		12 (63.2) 2 (10.5) 5 (26.3)	
Surgery:				
Radical Conservative Unknown	4 (23.5) 12 (70.5) 1 (5.9)	4 (80) 1(20)	9 (47.4) 10 (52.6)	2 (66.6) 1 (33.3)
Chemotherapy regimens:				
Antracyclines + taxanes Trastuzumab + taxane Carboplatin + taxane	15 (88.2) 2 (11.8) 0 (0)	0 (0) 0 (0) 5 (100)	16 (84.2) 3 (15.8) 0 (0)	0 (0) 0 (0) 3 (100)
State:				
Maiden Cohabitant Married Separate Divorced Widow	1 (5.9) 1 (5.9) 10 (58.8) 3 (17.6) 0 (0) 2 (11.8)	0 (0) 1 (20) 4 (80) 0 (0) 0 (0) –	1 (5.2) 2 (10.5) 15 (78.9) 1 (5.2) 0 (0) 0 (0)	0 (0) 0 (0) 2 (66.6) 0 (0) 0 (0) –
Schooling:				
Elementary school Middle school degree High school degree Master’s degree	1 (5.9) 1(5.9) 5 (29.4) 10 (58.8)	– – 3 (60.0) 2	1 (5.2) 2 8 8	– 1 (5.2) 1 (5.2) 1 (5.2)
Occupation:				
Employee Trader craftsman Freelance Housewife Unemployed Retired	14 0 (0) 1 (5.9) 1 (5.9) 0 (0) 1 (5.9)	3 (60) 0 (0) 0 (0) 1 (20) 0 (0) 1 (20)	6 1 (5.2) 4 7 (36.8) 0 (0) 1 (5.2)	1 (5.2) – 1 (5.2) – – 1 (5.2)

Patients presented predominantly breast cancer submitted to surgery (17 patients [77%] in the experimental arm and 19 [86%] patients in the control arm) with HER2-negative phenotype (15 patients [68%] in the experimental arm and 12 [55%] patients in the control arm). Patients had an ECOG status predominantly equal to 0 (**Table 2**), and the most common CT regimen was anthracyclines + taxanes. Most of the patients were married (50% in the VRE arm and 77% in the control arm), had a master’s degree (54% and 41% in VRE and control arm, respectively) and were employed (82% and 59% in VRE and control arm, respectively).

### 3.1 Psychological evaluations

#### 3.1.1 Psychological distress

The HADs mean scores were below the cut-off at both considered time points T1 and T3, underlining the absence, in the whole sample, of anxiety and depression (**Supplementary Table 1**).

Stratifying patients by distress severity according to scores, in the VRE arm, stability in normal scores was reported between T1 and T3 (55% vs 52%, respectively), along with an increase in patients with borderline scores (14% at T1 vs 33% at T3) and a decrease in patients with pathological scores (32% at T1 vs 14% at T3). In the control arm, a decrease in patients with normal scores (64% at T1 vs 50% at T3) and an increase in both patients with borderline (14% at T1 vs 20% at T3) and pathological (23% at T1 vs 30% at T3) scores was reported from T1 to T3 (**Supplementary Table 2**).

Considering depression, results showed a similar trend in the two arms with a reduction in normal scores (77% at T1 vs 57% at T3 in the VRE arm; 86% at T1 vs 70% at T3 in the control arm) and an increase in borderline (14% at T1 vs 29% at T3, VRE arm; 9% at T1 vs 20% at T3, control arm) and pathological scores (9% at T1 vs 14% at T3, VRE arm; 5% at T1 vs 10% at T3, control arm; **Supplementary Table 2**).

#### 3.1.2 Anxiety

**Figure 1:**
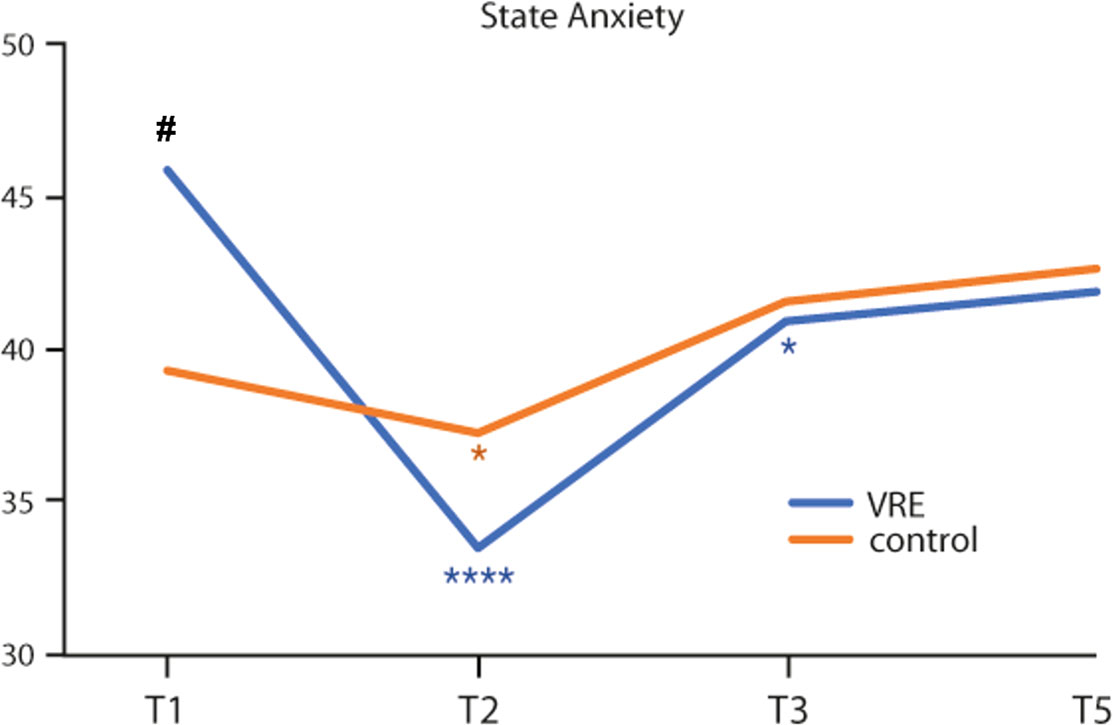
State anxiety mean values from the start to 48 hours from the end of the first chemotherapy cycle in the two study arms. *Intra-group variability. #Inter-group variability. Statistical significance: *^,#^p < 0.05; ****p < 0.0001.

In the VRE arm, a statistically significant reduction of the State Anxiety mean values was reported between T1 and T2 (45.9 ± 12.5 vs 33.4 ± 9.3, p<0.0001) and between T1 and T3 (45.9 ± 12.5 vs 40.9 ± 10.4, p=0.02). At T5, the observed mean value (41.9 ± 10.1) remains lower than the baseline value (**Figure 1**).

In addition, in the control arm, the State Anxiety mean values between T1 and T2 were statistically different (39.2 ± 9.5 vs 37.2 ± 9.0; p=0.04), albeit at a lower level. At T3 and T5, the observed mean values (41.6 ± 9.7 and 42.6 ± 10.8, respectively) tended to be higher than baseline (**Figure 1**).

Comparing the two groups, at T1 the State Anxiety was significantly higher in the VRE group than in the control group (45.9 ± 12.5 vs 39.2 ± 9.5; p=0.05) (**Figure 1**).

In the VRE group, at T1 there was a statistically significant difference between the Trait Anxiety (37.3 ± 8.6), and the State Anxiety (p=0.002) mean values. This difference was not statistically significant in the control group (Trait Anxiety: 40.6 ± 11.1; p=0.32). The Trait anxiety was not statistically different between the two groups (37.3 ± 8.6, VRE arm vs 40.6 ± 11.1, control arm; p=0.22).

### 3.2 Quality of life assessment

The mean scores related to the EORTC QLQ-C 30 questionnaire did not show statistically significant differences between the two groups considered time points, T1 and T3, except for constipation at T3 (p=0.02) (**Supplementary Table 3**).

### 3.3 Secondary endpoints

#### 3.3.1 Effective and perceived time of treatment

In the VRE arm, 86% (n=19) of patients reported the perception of a shorter duration of CT compared to the effective treatment time. The median perceived time was of 104 minutes (range: 99–105) versus a median of 141 minutes (range: 135–145) of real duration (p<0.0001).

In the control arm, 76% (n=16) of patients perceived a longer than the effective duration of CT (median perceived time: 170 minutes, range: 165–174; median real duration: 155 minutes, range: 150–160; p<0.004).

The median reported perceived time was statistically different between the two groups (p=0.02).

#### 3.3.2 Toxicity

All the enrolled patients were assessable for safety analysis. The most frequently reported treatment-related toxicities were mild to moderate (grades 1–2) (data not shown). The main toxicities reported after the first CT cycle were grade 2 alopecia and grade 3 and 4 neutropenia. They were similar in both arms (73% in the VRE arm vs 68% in the control arm for alopecia, 27% in both arms for neutropenia). Grade 3 emesis was less evident in the VRE arm than in the control arm, but not significantly (4% vs 14% respectively; p<0.24). In the VRE arm, asthenia was reported less than in the control arm (4% vs 36% respectively; p=0.008; **Table 3**).

**Table 3:** Grade 3 and 4 reported toxicities.

	VRE arm (n = 22), n (%)	Control arm (n = 22), n (%)
Alopecia (grade 2)	16 (72.7)	15 (68.1)
Neutropenia	6 (27.2)	6 (27.2)
Febrile neutropenia	2 (9.0)	4 (18.2)
Emesis	1 (4.5)	3 (13.6)
Hypertransaminasemia	1 (4.5)	1 (4.5)
Asthenia	1 (4.5)	8 (36.4)

#### 3.3.3 PRO data analysis

The patients’ perceptions investigated through the PROs at T4 (eight patients) and T5 (22 patients) showed a population with low toxicity. The statistical analysis between the time points considered was not studied for an imbalance of patients, so only T5 analysis was reported.

The analysis of the psychological variables included: insomnia (items 52), fatigue (items 53), anxiety (items 54), mood (items 55), sadness (items 56). At T5, all these were different between the VRE and control arm: severity and interference with usual or daily activities of insomnia [(9% vs 35% (p=0.04) and 14% vs 35% (p=not significant), respectively)]; a greater interference of the anxiety [(9% vs 35% (p=not significant), respectively)]; a perception of higher frequency and intensity in the mood [(14% vs 35% (p=not significant) and 0 vs 20% (p=0.03), respectively)]; a greater perception of interference in daily activities deriving from sadness (5% vs 14%, respectively); no difference was documented in fatigue item.

## 4 Discussion

In recent years, there has been an exponential development of VRE. Today, this entertainment intervention finds widespread application in various fields: from the fashion industry with digital dressing rooms to 360° VR photography, to the automotive sector and cinema, from the world of videogames to virtual museum visits. VR is not a new technology, but it is a tool that is going through radical evolution. There have been some advancements in industries with some interesting new possibilities. One of the most interesting and perspective-bending abilities of VR is the capability to immerse ourselves completely into an environment totally outside of our regular size, positioning us in a new relationship with the world. For instance, the growing field of immersive microscopy is putting doctors and scientists into microscopic worlds, giving them literal new perspectives of what is happening inside the human body, down to the scale of connected networks of neurons within the brain structure. Being able to visualize and manipulate the world at this scale holds incredible possibilities for solving medical problems, the potential of which has generated a lot of interest in the field from outside investors.

For the reason stated above, also in health, VRE is gaining increasing interest, finding potential areas for development and application in the field of diagnostics, therapy, training and prevention.

In breast and gynecological patients, psychological distress is present at diagnosis and after surgery (11). The first experiments to assess the impact of VRE in cancer patients undergoing CT dates back to 1999 (12). The results of the seven randomized clinical trials published from 1999 to 2011 supporting the use of the VRE reported a reduction of the symptoms related to distress, fatigue, anticipatory nausea, and perception of the duration time of CT administration (13).

Although encouraging, these results derive mainly from pilot studies with low or mixed samples and limited statistical power. They explored various relevant variables, including different settings (i.e., during chemotherapy, during painful procedures, during hospitalization, and during port access). Moreover, most were the result of experiments conducted by technology and contents are now considered obsolete. Specifically, at that time, most VREs were limited to graphic reconstruction of reality without further direct experience with VRE, highlighting the need for more modern and innovative technologies.

In the present study, 44 breast and ovarian cancer patients were randomly assigned to receive VRE or to entertain themselves with conventional means during the first cycle of adjuvant CT, with the aim to evaluate if patients in the VRE arm reported an early improvement of psychological, anxiety, and quality of life outcomes. In this study, thanks to applying the most modern technologies, it was possible to give a “dream” during adjuvant CT exposure, allowing patients to view and live an immersive global experience.

Results from HAD questionnaire underlined the absence of prevalent disturbs of anxiety and depression in both groups.

A prevalence of normal scores related to depression was reported in both groups, along with a similar trend over time. This result underlines the low impact of VRE on the depressive state.

Regarding anxiety, our findings showed a different distribution of scores over time. In the comparison between T1 and T3 in the VRE arm, there was an increase in patients with borderline scores and a decrease in patients with pathological scores. On the contrary, in the control group, an increase in both borderline and pathological scores were observed at T3.

This trend suggests the impact of VRE on the anxiety outcome, as supported by data relating to state anxiety. Indeed, even if the state anxiety values before and after the first CT cycle decreased in both groups, this reduction is statistically significant over time only in the VRE arm. This difference in the scoring trend could be related to the different scores in trait anxiety, which tend to be lower in the VRE group, indicating a population whose anxiety is predominantly situational, determined by a stressful event such as CT. VRE would therefore act more positively in a population that presents an increased state of anxiety related to the crisis event in the absence of an anxious basic structure.

QoL scores related to the EORTC QLQ-C 30 questionnaire overall compared the two study groups.

The duration of therapy perceived by patients undergoing distraction therapy was significantly shorter when compared to the control group, which reports a perception of time greater than the real duration of therapy.

Regarding toxicity data, the use of VRE during the first CT cycle appears to reduce asthenia outcomes. The PROs results analyzed at T5 were in line with the results of the STAI scale, confirming the presence of a better psychological state in the patients of the VRE group. Taken together, these data suggest that the VRE positively influenced the levels of state anxiety among patients. Moreover, even if a cost analysis goes beyond the scope of this study, we can speculate that a VRE intervention could reduce the costs related to drug therapy and support interventions for the anxiety management.

Our results were in line with other previous studies about the impact of VRE on the health system and during cancer treatments. These studies found that VRE improved patients’ emotional well-being and diminished cancer-related psychological symptoms (4, 13–17) in different settings. Nevertheless, the time of the VRE exposition was very short in most of these experiences. VRE’s impact on clinical variables involved in distress (pulse rate, blood pressure) has been investigated only partially. In this context, our study is the first to evaluate the use of VRE during the first cycle of CT in breast or ovarian cancer patients, thus analyzing a specific homogeneous subgroup of patients and with a methodologically improved study design. In addition, our study used a relatively high-tech VRE with highly interactive virtual worlds. Considering that immersion and interactivity impact VRE efficacy, stronger results might likely be obtained with more immersive and fully interactive experiences. In addition, few studies compared the efficacy of VRE with a concurrently randomized control group and, therefore, are at risk of bias.

Our study presents some limitations, mainly related to the sample’s small size and the short-term analysis. In addition, from our data, it was not possible to identify the mechanism of action of VRE on anxiety nor the population that could benefit most from this strategy. Further studies are therefore needed to define the role of VRE in improving the psychological well-being of patients undergoing CT. This will allow virtual reality to be used more effectively in daily clinical practice.

## Conclusion

In the era in which the quality of life of cancer patients is taking a fundamental role, it is a primary goal to improve the benefit of the cure and the life of long survival patients. Our study suggests that the use of VRE has some benefits on the state anxiety of the first cycle of CT. Since the first cycle of CT can not scan impact subsequent cycles, not only for toxicity related to treatment but also for emotional distress, this tool could also be useful for a more important acceptance of the treatment and compliance with the therapy.

Our results support the continuous research on VRE as a distraction intervention, with the aim to meet the clinical need for effective nonpharmacologic adjunctive therapies.

## Data availability statement

The original contributions presented in the study are included in the article/**Supplementary Material**. Further inquiries can be directed to the corresponding author.

## Ethics statement

The studies involving human participants were reviewed and approved by Central Ethics Committee (protocol registration number: RS 1105/18). The patients/participants provided their written informed consent to participate in this study.

## Author contributions

Study design: AF, FG, DG. data collection and interpretation: All. manuscript writing: AF, VR, and CF. manuscript editing: All. All authors contributed to the article and approved the submitted version.

## Acknowledgments

Editorial assistance was provided by Simonetta Papa, PhD, Massimiliano Pianta and Aashni Shah (Polistudium SRL, Milan, Italy). This assistance was supported by internal funds.

## Conflict of interest

Authors FG, AG and GG are funders of Twiceout.

The remaining authors declare that the research was conducted in the absence of any commercial or financial relationships that could be construed as a potential conflict of interest.

## Publisher’s note

All claims expressed in this article are solely those of the authors and do not necessarily represent those of their affiliated organizations, or those of the publisher, the editors and the reviewers. Any product that may be evaluated in this article, or claim that may be made by its manufacturer, is not guaranteed or endorsed by the publisher.
