# Supplementary material for: The immersive experience of virtual reality during chemotherapy in patients with early breast and ovarian cancers: The patient’s dream study
Source: Front Oncol. 2022 Sep 30;12:960387. doi: 10.3389/fonc.2022.960387 (PMC9563848; doi:10.3389/fonc.2022.960387)
Supplement: Supplementary file 1 [file Table_1.docx]

**The immersive experience of virtual reality during chemotherapy in patients with early breast and gynecological cancers: the Patient’s Dream study**

| **Supplementary Table 1. Psychological distress evaluations** | | | | | | |
| --- | --- | --- | --- | --- | --- | --- |
| **HADs** | **T1 (mean±SD)** | | | **T3 (mean±SD)** | | |
|  | **VRE group** | **Control group** | **p-value** | **VRE group** | **Control group** | **p-value** |
| Anxiety* | 6.8±3.8 | 6.9±4.4 | 0.95 | 6.9±3.5 | 7.9±4.2 | 0.37 |
| Depression** | 5.1±3.1 | 4.3±3.2 | 0.42 | 6.4±3.9 | 5.6±3.4 | 0.50 |
| HAD: Hospital Anxiety and Depression scales; VRE: virtual reality experience.  *Repeated measure ANOVA test: p=0.48.  ** Repeated measure ANOVA test: p=0.53. | | | | | | |

| **Supplementary Table 2. Psychological distress evaluations according to the stratification proposed by Carrol et al [Carrol 1993].** | | | | | | |
| --- | --- | --- | --- | --- | --- | --- |
| **HADs** | **T1, n (%)** | | | **T3, n (%)** | | |
|  | **VRE group** | **Control group** | **p-value** | **VRE group** | **Control group** | **p-value** |
| Anxiety*:   - ≤7 - 8–10 - ≥11 | 12 (54.5)  3 (13.6)  7 (31.8) | 14 (63.6)  3 (13.6)  5 (22.7) | 0.78 | 11 (52.4)  7 (33.3)  3 (14.3) | 10 (50.0)  4 (20.0)  6 (30.0) | 0.40 |
| Depression*:   - ≤7 - 8–10 - ≥11 | 17 (77.3)  3 (13.6)  2 (9.1) | 19 (86.4)  2 (9.1)  1 (4.5) | 0.73 | 12 (57.1)  6 (28.6)  3 (14.3) | 14 (70.0)  4 (20.0)  2 (10.0) | 0.69 |
| HAD: Hospital Anxiety and Depression scales; VRE: virtual reality experience.  *≤7: normal condition; 8-10: borderline case; ≥11: clinical cases | | | | | | |

| **Supplementary Table 3. Quality of life assessment.** | | | | | | |
| --- | --- | --- | --- | --- | --- | --- |
| **Items** | **T1 (mean±SD)** | | | **T3 (mean±SD)** | | |
|  | **VRE group** | **Control group** | **p-value** | **VRE group** | **Control group** | **p-value** |
| Global C30 | 71.5±14.8 | 68.6±19.6 | 0.58 | 58.4±24.6 | 55.1±21.6 | 0.65 |
| Physical C30 | 94.5±6.0 | 89.9±11.0 | 0.09 | 76.1±22.8 | 74.7±16.7 | 0.81 |
| Role C30 | 86.4±15.9 | 90.1±15.1 | 0.42 | 73.0±29.5 | 80.0±18.3 | 0.37 |
| Emotional C30 | 73.8±16.9 | 66.7±20.0 | 0.21 | 77.0±16.9 | 68.3±24.0 | 0.19 |
| Cognitive C30 | 87.8±18.7 | 81.0±27.4 | 0.34 | 84.9±19.6 | 80.8±23.1 | 0.54 |
| Social C30 | 82.6±19.5 | 84.1±22.7 | 0.82 | 73.9±24.3 | 76.7±21.2 | 0.70 |
| Fatigue C30 | 16.5±15.5 | 16.0±16.9 | 0.92 | 38.4±30.0 | 34.8±18.8 | 0.64 |
| Nausea and vomiting C30 | 2.3±7.8 | 5.3±10.7 | 0.29 | 20.4±26.4 | 21.8±14.3 | 0.84 |
| Pain C30 | 9.9±15.1 | 11.5±16.6 | 0.74 | 21.4±28.4 | 20.1±19.9 | 0.86 |
| Dyspnea C30 | 6.0±13.0 | 9.0±15.0 | 0.48 | 15.8±24.9 | 16.6±20.2 | 0.91 |
| Insomnia C30 | 22.6±31.5 | 34.7±30.0 | 0.20 | 32.2±35.3 | 34.9±23.0 | 0.77 |
| Appetite loss C30 | 13.6±26.5 | 7.6±17.6 | 0.38 | 25.4±27.8 | 23.3±24.5 | 0.80 |
| Constipation C30 | 16.6±26.7 | 12.1±26.3 | 0.58 | 30.1±29.7 | 10.0±22.0 | **0.02** |
| Diarrhea C30 | 6.0±13.0 | 6.0±13.0 | 0.99 | 4.7±11.8 | 9.2±18.3 | 0.36 |
| Financial difficulty C30 | 9.1±23.4 | 19.6±35.1 | 0.25 | 9.5±18.7 | 23.3±36.0 | 0.13 |
| VRE: virtual reality experience.  Bold valuies indicate significant difference. | | | | | | |
